# Host–Guest Chemistry as a Supramolecular Engine for Iontronic Transduction in Nanochannels

**DOI:** 10.3390/molecules31040713

**Published:** 2026-02-19

**Authors:** L. Miguel Hernández Parra, Angel L. Huamani, Ignacio T. Matelo, M. Lorena Cortez, Matías Rafti, Gregorio Laucirica, Waldemar Marmisollé, Omar Azzaroni

**Affiliations:** 1 Instituto de Investigaciones Fisicoquímicas Teóricas y Aplicadas (INIFTA), Departamento de Química, Facultad de Ciencias Exactas, Universidad Nacional de La Plata (UNLP), CONICET, Boulevard 113 y 64, La Plata 1900, Argentinaignaciomatelo@inifta.unlp.edu.ar (I.T.M.); wmarmi@inifta.unlp.edu.ar (W.M.); 2 GSI Helmholtzzentrum für Schwerionenforschung, 64291 Darmstadt, Germany

**Keywords:** host–guest chemistry, iontronic, nanochannels, stimuli-response

## Abstract

Since the first synthetic macrocyclic receptors were shown to bind ions selectively, supramolecular host–guest chemistry has enabled the translation of molecular recognition events into physical signals. Early coupling of such receptors to ion-sensitive field-effect transistors established a bridge between supramolecular chemistry and solid-state electronics. Today, this bridge is rebuilt in iontronics, where ions carry information through nanoconfined media and ionic transport becomes highly sensitive to electrostatic gradients, surface charge, and surface molecular interactions. As a result, ionic flux can serve as an efficient transduction mechanism that responds precisely, reversibly, and rapidly to changes in the chemical environment. Within this regime, host–guest chemistry offers a powerful means to exert direct control over ionic behavior, allowing molecular recognition to modulate conductance, rectification, and ion selectivity, thereby conferring practical function to nanofluidic systems. This review highlights systems in which host molecules act as chemical actuators that modulate nanochannel surface chemistry, thereby regulating ionic flux and enabling reversible, tunable, and stimulus-responsive behaviors. We survey architectures in which crown ethers, calixcrowns, pillararenes, and related hosts are integrated into solid-state nanochannels, emphasizing representative achievements such as biological-level Na^+^/K^+^ selectivity in crown ether-based systems and nanomolar-level detection of ions using calixcrowns- and pillararene-functionalized nanochannels. Finally, we discuss how temperature, pH, light, and redox state act as external stimuli that reversibly switch between conductive states, yielding ion-selective platforms for sensing and ion sieving.

## 1. Introduction

The translation of molecular recognition events into measurable physical signals has been a central pursuit of supramolecular chemistry since the pioneering works of Pedersen [1], Cram [2], and Lehn [3]. Their discovery that synthetic macrocyclic receptors—such as crown ethers and cryptands—could bind ions and small molecules with high selectivity laid the foundation for molecular information processing. From this moment, chemistry began to encode function into interactions rather than covalent structures, establishing the basis for converting host–guest phenomena into transduced outputs.

During the 1980s, Reinhoudt and colleagues [4] extended this vision into the technological realm by integrating host–guest recognition with field-effect transistors (FETs). The resulting ion-sensitive and chemically sensitive FETs (ISFETs and CHEMFETs) demonstrated how molecular binding events could modulate the electrical potential at semiconductor interfaces, producing measurable changes in current. This connection between supramolecular chemistry and solid-state electronics represented one of the earliest conceptual bridges linking molecular recognition to signal transduction.

Today, that bridge is being reconstructed within a different physical regime: the realm of iontronics [5]. Unlike traditional electronics, which rely on electrons in semiconductors, iontronics exploits ions as mobile information carriers in confined, soft, and hydrated environments. Ionic transport through nanofluidic architectures—such as nanochannels—is governed by electrostatic potential gradients, charge selectivity, and molecular interactions, all of which are highly sensitive to the local chemical environment [6]. Within this emerging framework, host–guest systems offer a new level of control: molecular recognition can now be coupled directly to ionic transport phenomena, producing devices that convert chemical information into dynamic ionic signals.

Nanofluidic diodes exemplify this transformation. By exploiting asymmetry in charge distribution, geometry, or chemical functionalization, these systems achieve rectification, gating, and amplification behaviors analogous to those of electronic components [7,8]. However, the underlying physics is entirely different: current results from ion transport rather than electron flow, and the signal arises from modulation of ionic conductance and device selectivity.

The convergence of supramolecular chemistry and iontronics thus enables a new type of signal pathway—one in which molecular recognition is intrinsic to transport rather than peripheral. Host molecules such as crown ethers, cucurbiturils, or calixarenes can act as “ion traps” [9], controlling the behavior of ionic species within nanoconfined electrolytes. When embedded into nanofluidic architectures, these receptors alter local ion densities and potential landscapes, effectively coupling host–guest binding equilibria to measurable current–voltage characteristics. This “chemical-to-iontronic” transduction represents a frontier in molecular actuation, sensing, and even computation, enabling devices that process information through the “language” of ions rather than electrons [10].

## 2. From Electronic to Iontronic Transduction: A Paradigm Shift

The shift from electronic to iontronic transduction entails a profound conceptual transition. While ISFETs demonstrated that supramolecular recognition could alter surface potentials at oxide-semiconductor interfaces, modulating the density of charge carriers in the transistor channel, iontronic systems transpose this logic into electrolytic environments where ions serve as the active charge carriers. Whereas electrons or holes in solids exhibit high mobility, ballistic transport in nanostructures, and well-defined energy bands that enable fast, low-noise responses, solvated ions move more slowly through liquids, gels, or hydrated polymers. Their transport is strongly coupled to electrostatic screening, electrical double layer formation, and surface interactions, fundamentally changing the rules for signal transduction [11]. The slower dynamics of ionic processes can, in fact, be advantageous. Ions in nanometric channels do not immediately return to their initial distribution, often stabilizing metastable configurations that can encode memory or temporal filtering. In nanofluidic enzymatic biosensors, for instance, enzymatic reactions modify the confined ionic environment, producing stable output signals that persist over time despite ongoing concentration gradients [12]. Another example refers to devices whose response depends on the history of the stimulus, especially in the case of ion accumulation due to preconfigured polarization of the nanofluidic architecture. In such architectures, ion-based memristive behaviors can emerge, where conductance effectively stores the voltage history [13,14].

The combination of confinement, electrical double layer overlap, and concentration polarization naturally gives rise to nonlinear behaviors, including rectification [15], hysteresis, and bistability [16], which amplify subtle chemical changes. In extremely narrow channels, transport becomes highly dependent on geometry and size, so small variations in surface charge or ion concentration produce large current changes [17]. In channels approaching the Debye length, overlapping double layers make ionic currents nonlinearly sensitive to local chemical modifications, allowing a single molecular binding event to generate a substantial change in current [18,19]. Similarly, concentration polarization under strong electric fields induces ion accumulation and depletion, giving rise to diode-like rectification, voltage-triggered transitions, and even switching instabilities [13]. A minor perturbation in local ion concentration can shift the system between distinct conductive states, providing natural amplification for sensing applications.

Beyond these physical effects, the chemical nature of ions introduces additional degrees of freedom that can be harnessed as functional “control knobs”. Ions can bind selectively to surfaces or participate in redox or enzymatic reactions. Specific adsorption can modulate surface charge and ionic selectivity, effectively acting as a gate that tunes ionic current, while complexation equilibria enable threshold-dependent conduction and chemical logic reminiscent of receptor-mediated signaling in biological membranes. Surface-mediated reactions can generate or consume ionic species, modify local pH, or even produce new species with distinct transport properties, actively driving iontronic functionality. For example, enzymatic production of hydroxide ions in the presence of Ca^2+^ can trigger precipitation of calcium hydroxide within a channel, abruptly reducing conductivity and generating a detectable signal [19].

Altogether, these effects illustrate a fundamental conceptual shift: from modulating electron flow through solid materials to controlling ion flow in dynamic, soft environments. In this context, chemical gating emerges naturally as molecular recognition events modify effective surface charge or the accessible ion area, tuning nanochannel conductance [20]. Even though the term “molecular recognition” evokes a wide variety of chemical processes, in our case this term refers to a scenario in which the ionic transport can be controlled with accuracy and convenience depending on the chemical entity that we integrate into the device. Exciting opportunities are revealed when we think in this manner, especially when host–guest interactions can function as reversible switches that gate ionic current, opening new avenues for amplification, logic, and sensing. Importantly, iontronics does not replace electronics but complements it, extending information processing into media compatible with soft matter. In this way, nanofluidic channels become platforms where supramolecular design and interfacial physical chemistry converge, enabling responsive devices operating at the intersection of chemistry, materials science, and nanofluidics.

In the following sections, host–guest architectures are first presented according to host type, followed by an examination of how external stimuli can dynamically modulate their function, thereby enabling external control over ionic flux and ion selectivity. Although this review focuses primarily on single track-etched nanochannels, it is important to mention that studies on other nanofluidic architectures (e.g., 2D nanoporous materials, nanoporous membranes, multichannel arrays, and glass nanopipettes) also provide valuable insight into host–guest chemistry under nanoconfinement. Detailed descriptions of the fabrication and performance of other platforms are available in the literature, and the reader is referred to the following references for further information on these systems [21,22,23,24,25,26].

### 2.1. Host–Guest Supramolecular Architectures in Artificial Nanochannels

Single-pore nanochannels are typically fabricated in polymer membranes using different techniques, with ion-track etching being among the most widely employed methods [27], because it enables precise control over pore density (from single-pore devices to multipore membranes), as well as pore size and shape. Briefly, the process consists of irradiating the polymer with high-energy heavy ions to generate latent tracks, followed by a controlled chemical etching step that develops the pore and allows its geometry to be tailored by adjusting the etching conditions [28].

These nanochannels provide a versatile platform to investigate transport phenomena under confinement while minimizing ensemble-averaging effects and pore interactions inherent to multipore membranes, thereby helping to isolate the phenomenon under study [29,30]. This, in turn, facilitates correlating variations in the iontronic response with well-defined changes within the channel, and particularly in surface characteristics such as the ionic environment, effective charge and interfacial chemistry. Such control is particularly valuable for studying host–guest interactions in confined spaces [31], where, as discussed above, binding events at the interface can be translated into iontronic response changes. In this context, continuum modeling based on Poisson–Nernst–Planck (PNP) theory is often used, due to the relative simplicity of modeling a single nanochannel to rationalize I–V characteristics and link experimental observables to key physicochemical parameters [32].

The host–guest architectures share several structural features. Most hosts are built from preorganized cyclic or polycyclic scaffolds (such as crown ethers, calixarene-crown hybrids, pillar[n]arenes, or cyclen-derived chelators like 1,4,7,10-tetraazacyclododecane-1,4,7-triacetic acid -DO3A-) which define well-shaped cavities that can be covalently anchored at the nanochannel wall. These frameworks are typically rich in oxygen and/or nitrogen donor atoms (ether oxygens, phenoxy groups, amines, and carboxylates), providing arrays of coordination and hydrogen bonding sites that bind ions or molecules with high affinity and selectivity. Despite their chemical diversity, they all operate on the same principle: reversible complexation at these heteroatom-rich cavities is converted into controllable changes in local charge, hydration, and effective pore size, thereby gating ionic transport through the nanochannel.

#### 2.1.1. Crown Ethers

**Figure 1:**
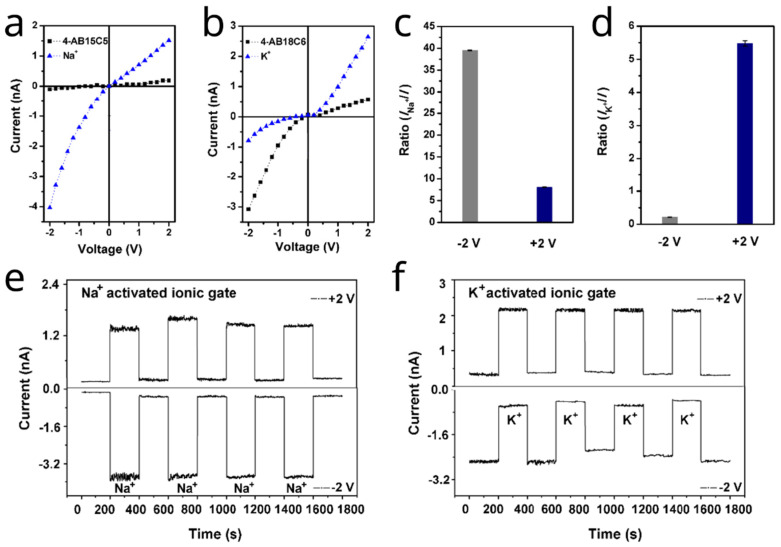
(**a**,**b**) I-V curves of 4-AB15C5 and 4-AB18C6 nanochannels before and after exposure to Na^+^ and K^+^, respectively. (**c**,**d**) Current ratio at different voltages. (**e**,**f**) Chronoamperometric responses in the presence of the corresponding ionic guest. Adapted from Ref. [33].

One of the earliest studies employing host–guest chemistry to manipulate nanofluidic transport properties was conducted by Lei Jiang and collaborators. These researchers utilized conical polyimide (PI) nanochannels that exhibit intrinsic ionic rectification due to their asymmetric geometry, enabling selective ion transport. Inspired by biological ion channels, the study focused on creating sodium (Na^+^)- and potassium (K^+^)-activated nanochannels by immobilizing alkali metal cation-responsive crown ethers—4′-aminobenzo-15-crown-5 (4-AB15C5) and 4′-aminobenzo-18-crown-6 (4-AB18C6)—onto the PI nanochannels. Crown ethers were selected for their strong selectivity, facile functionalization, and compatibility with alkali metal cations. The cavity size of 15-crown-5 matches that of Na^+^ (0.98 Å), while 18-crown-6 corresponds to K^+^ (1.38 Å). In Na^+^-activated ionic gates, the 4-AB15C5-modified nanochannels switch between “off” and “on” states upon Na^+^ exposure (Figure 1a–d).

In the presence of Na^+^ ions, the current increases significantly (from −0.1 nA to −4.2 nA at −2.0 V and from +0.2 nA to +1.6 nA at +2.0 V) due to altered surface charge, wettability, and effective pore size. A similar trend is observed in K^+^-activated ionic gates, where 4-AB18C6-modified nanochannels initially display selective cation conduction and, following K^+^ activation, exhibit current reversal that facilitates selective anion conduction. Association constants determined by ^1^H NMR titration were 13.2 M^−1^ for 4-AB15C5/Na^+^ and 23.3 M^−1^ for 4-AB18C6/K^+^. The stronger binding of K^+^ accounts for the more pronounced positive surface charge and current inversion. This work demonstrates the successful development of Na^+^- and K^+^-responsive ionic gates with high sensitivity, selectivity, stability, and reversible switchability (Figure 1e,f) [33].

**Figure 2:**
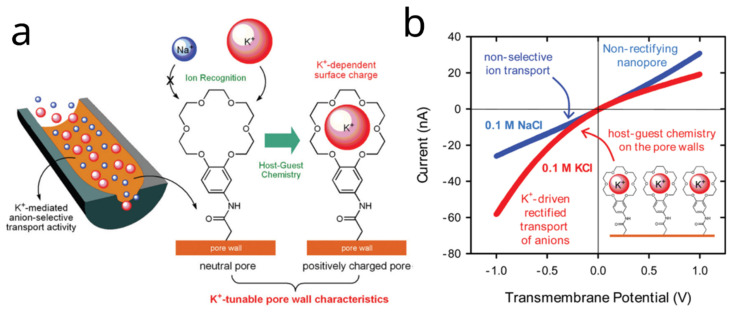
K^+^-activated ionic gating via host–guest chemistry. (**a**) Scheme of K^+^ binding to crown ether groups on the pore wall, altering the effective surface charge. (**b**) I–V curves showing non-rectifying transport in 0.1 M NaCl and K^+^-induced rectification in 0.1 M KCl. Adapted from Ref. [31].

Another early exploration of host–guest chemistry employed asymmetric bullet-shape nanopores modified with 18-crown-6 moieties to fabricate a nanodevice capable of modulating ionic transport based on potassium ion concentration. The crown ether-modified nanopores exhibit specific affinity toward K^+^, inducing surface charge alterations on the pore walls. This interaction allows dynamic control of ionic transport and rectification behavior, mimicking biological ion channels. In the presence of K^+^, the nanopore surface transitions from neutral to positively charged, resulting in anion-selective transport. By contrast, Na^+^ does not interact significantly with the crown ether units, leaving the nanopores non-rectifying (Figure 2). By tailoring the derivatization time, a mixture of carboxylate and crown ether groups remains on the pore surface, enabling modulated rectified transport depending on environmental K^+^ concentrations. PNP modeling and experimental results revealed effective surface charge densities in the presence of various alkali ions—K^+^ (0.1 e nm^−2^), Na^+^ (0.25 e nm^−2^), Rb^+^ (0.275 e nm^−2^), Cs^+^ (0.5 e nm^−2^), and Li^+^ (0.5 e nm^−2^)—consistent with the highest known affinity of 18-crown-6 for K^+^ ions [31].

Remarkably, using a closely related strategy and channel architecture, Wang and co-workers [34] demonstrated that potassium ions can also be released from the 18-crown-6 anchored on the channel surface upon application of a sufficiently high transmembrane potential (+3 V). This observation indicates that, beyond the host–guest-induced modulation of surface charge and the associated changes in ion transport, the nanofluidic device can also operate as a voltage-gated platform.

**Figure 3:**
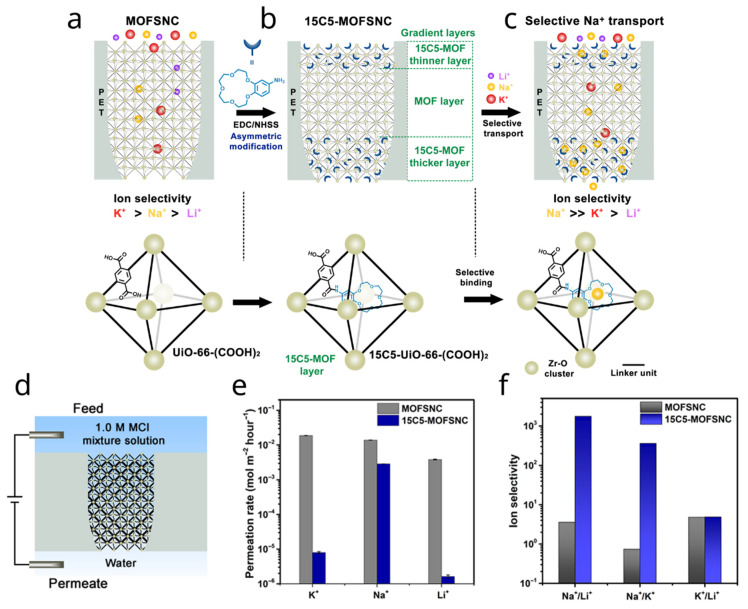
(**a**–**c**) Stepwise modification of the MOF-modified nanochannel to incorporate the Na^+^-selective crown ether. (**d**) Permeation experiment setup. (**e**) Ion permeation before and after crown ether modification. (**f**) Calculated selectivity. Adapted from Ref. [35].

Building upon these early examples of crown-ether-modified nanopores, Jiang and co-workers recently translated crown-ether-based molecular recognition into a rigid Metal–Organic Framework (MOF)-confined nanochannel architecture [35]. They first grew UiO-66-(COOH)_2_ Zr-based MOF inside single bullet-shaped PET nanochannels, creating an angstrom-scale MOF subnanochannel (MOFSNC) that homogeneously fills the pore. A subsequent post-synthetic amidation between 4′-aminobenzo-15-crown-5 and the pendant carboxylic groups of the linker yields a heterostructure in the axial direction, where crown ethers are mainly confined near the tip and base regions while only a small fraction penetrates the central MOF zone (Figure 3a–c).

In the pristine UiO-66-(COOH)_2_-filled nanochannels, the MOF scaffold alone exhibits a transport preference for K^+^ over Na^+^ and Li^+^, arising from a combination of size exclusion and framework-ion affinity. Introducing the 4-AB15C5 crown ether at the channel entrances converts this intrinsic preference into an artificial sodium-selective channel. Under multicomponent transport conditions, the resulting device exhibits Na^+^ ≫ K^+^ > Li^+^ selectivity, with Na^+^/K^+^ selectivity factors in the range of 10^1^ to 10^2^, and Na^+^/Li^+^ values reaching 10^3^, approaching those reported for biological sodium channels (Figure 3d–f) [36].

#### 2.1.2. Calixcrowns

**Figure 4:**
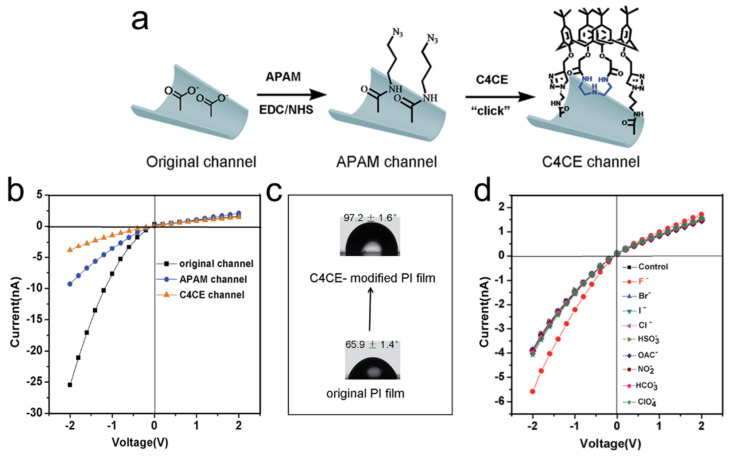
(**a**) Construction of the single conical nanochannel for F^−^ sensing. (**b**) I–V curves after each modification step. (**c**) Contact angle on the flat film before and after C4CE modification. (**d**) I–V curves of the C4CE-modified nanochannel in 0.1 M KCl and 0.05 M Tris–HCl (pH 7.0) in the presence of 1 mM F^−^, Cl^−^, Br^−^, I^−^, HSO_3_^−^, OAc^−^, NO_2_^−^, HCO_3_^−^, and ClO_4_^−^. Adapted from Ref. [37].

A similar approach was employed to construct nanofluidic devices sensitive to fluoride ions. In this case, nanochannels were derivatized with 1,3-dipropargylaza-p-tert-butyl calix[4]crown (C4CE) via click chemistry to achieve selective detection of fluoride (F^−^) in aqueous media, including complex biological matrices such as serum. Fluoride plays critical roles in health but becomes toxic when accumulated excessively. Existing sensors often require organic media and suffer from poor selectivity. Nie et al. fabricated a conical nanochannel functionalized with C4CE, containing -NH- groups that bind fluoride selectively through hydrogen bonding [37]. The device demonstrated strong selectivity over competitive anions (Cl^−^, Br^−^, I^−^, HSO_3_^−^, OAc^−^, NO_2_^−^, HCO_3_^−^, ClO_4_^−^), with a binding constant of 1.17 × 10^6^ M^−1^. A detection limit of 9.7 × 10^−7^ M was achieved, with measurable current changes even at 10^−9^ M F^−^. Spectroscopic analyses (^1^H NMR, ^19^F NMR, XPS, LSCM) confirmed hydrogen bonding interactions (Figure 4). The system also exhibited recyclability through fluoride removal by Ca^2+^ complexation. The device successfully detected fluoride in serum with a recovery rate of 79.8% [37].

Responsive nanochannels for cesium detection have been developed by functionalizing conical nanopores with amine-terminated p-tert-butylcalix[4]arene-crown (t-BuC[4]C-NH2) moieties. These calixcrowns selectively bind Cs+ through oxygen coordination and cation–π interactions, imparting positive fixed charge that switches the nanochannel from a nonconductive “off” state to a conductive “on” state. The current increased with Cs^+^ concentration, reaching 1.66 nA at −2 V in 100 mM CsCl. A detection threshold of 100 μM was reported with reliable performance reproducibility [38].

#### 2.1.3. Pillararenes

Zhang et al. utilized mercaptoacetic acid pillar[5]arene (MAP5) assembled on conical nanochannels to develop Hg^2+^-sensitive nanofluidic devices [39]. MAP5 forms host–guest complexes with 1,6-hexanediamine (HDA), enabling reversible switching between “on” (high current) and “off” (low current) states. Hg^2+^ has a stronger affinity for MAP5 and displaces HDA, modulating surface charge and conductance. The system exhibited high selectivity and a 1 nM detection limit, with current decreasing from 4.8 nA to 0.5 nA at 2 V upon Hg^2+^ exposure. This biomimetic design mimics mercury-induced potassium channel blockage and offers insight into mercury toxicity mechanisms [39].

**Figure 5:**
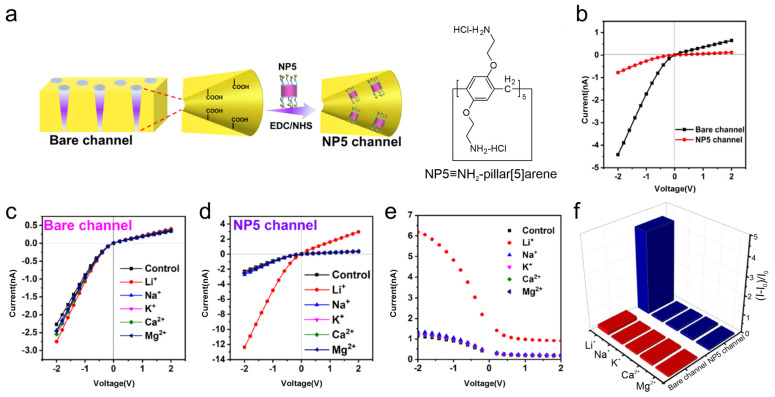
(**a**) Scheme of nanochannel modification with NH_2_-pillar[5]arene (NP5) via the EDC/NHS route and structure of NP5. (**b**) I-V curves before and after NP5 modification. (**c**) Control experiment: unmodified nanochannel response to different ions. (**d**) Response of the NP5-modified membrane to different ions. (**e**) Extended I-V curves (−2 to 2 V) for the same ions. (**f**) Relative rectification factor for different ions with respect to the control. Adapted from Ref. [40].

This framework has also been adapted to Li^+^-responsive nanochannels using NH_2_-pillar[5]arene (NP5), which forms strong cooperative host–guest interactions with LiCl. Chloride binds to NP5 via hydrogen bonding, promoting Li^+^ association through cation–π interactions. NP5 demonstrated high selectivity for LiCl with an association constant of 336.6 ± 46.3 M^−1^, outperforming NaCl, KCl, CaCl_2_, and MgCl_2_ (Figure 5) [40].

#### 2.1.4. Other Architectures

**Figure 6:**
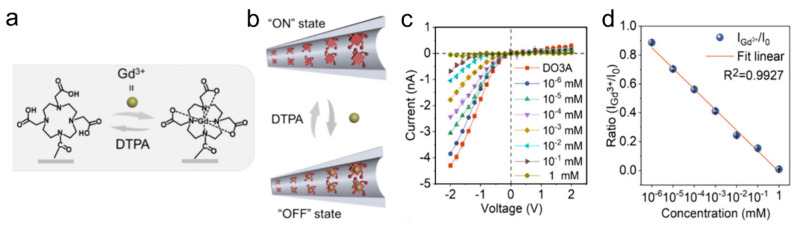
(**a**) Gd^3+^ coordination by DO3A and reversal using DTPA. (**b**) Schematic of Gd^3+^-triggered ON/OFF switching in the nanochannel. (**c**) I–V curves at different Gd^+3^ concentrations. (**d**) Normalized current ratio as a function of Gd^+3^ concentration (linear fit). Adapted from Ref. [41].

Beyond the macrocyclic cavity hosts discussed above, host–guest regulation of nanofluidic transport can also be achieved using coordination chelators such as DO3A. Inspired by Gd^3+^-induced ionic blockade in biological channels, Sun et al. fabricated DO3A-modified nanochannels showing ultrasensitive Gd^3+^ responsiveness [41]. Gd^3+^ binding produces dramatic conductance decreases (up to 100-fold at 1 mM) and cation-to-anion selectivity reversal. Binding reversibility using the strong chelator diethylenetriaminepentaacetic acid (DTPA) enabled reversible switching and multilevel information encryption using multiple conductive states. A portable encryption device incorporating DO3A nanochannel membranes was successfully demonstrated (Figure 6) [41].

### 2.2. Stimuli-Responsive Modulation

While the intrinsic affinity and selectivity of these host–guest architectures are largely encoded in their molecular design, they can be further modulated by external stimuli such as pH, light, temperature, or redox conditions. In this way, binding events become gated rather than static, enabling nanochannels that behave as “smart” iontronic devices, whose transport properties can be dynamically switched or reprogrammed in response to their chemical or physical environment.

#### 2.2.1. pH

**Figure 7:**
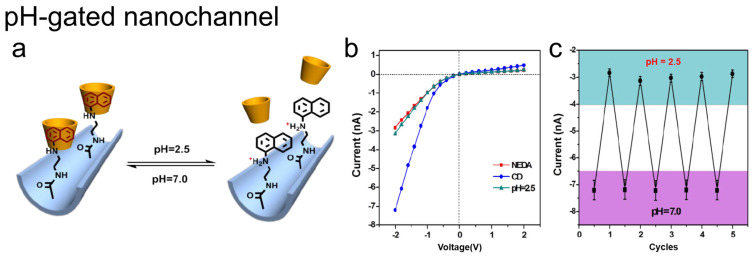
pH-gated nanochannel based on reversible γ-CD/NEDA host–guest assembly. (**a**) Schematic of γ-CD inclusion on a NEDA-functionalized nanochannel at pH 7.0 and disassembly upon acidification (pH 2.5). (**b**) I–V curves at different stages of the pH-controlled assembly/disassembly process. (**c**) Reversible current switching over repeated pH cycles (pH 2.5 ↔ 7.0). Adapted from Ref. [43].

Host–guest chemistry within nanochannels has also been applied to pH-responsive transport control. Li and co-workers constructed a molecular switch based on reversible complexation between N-acetyl-cysteine pillar[5]arene (ACP5) and HDA. Proton-driven conversion between carboxylate and neutral carboxylic acid groups modulates threading/dethreading within the confined nanochannel environment, enabling reversible pH-dependent gating [42]. This pH reversibility can also be achieved using cyclodextrin-based host–guest pairs. Li’s group assembled γ-cyclodextrin (γ-CD) onto a N-(1-naphthyl)ethylenediamine (NEDA)-functionalized nanochannel via inclusion of the naphthyl guest at pH 7.0. Upon acidification (pH = 2.5), protonation of NEDA weakens the host–guest interaction and promotes disassembly, restoring the initial transport state (Figure 7a,b). Repeated pH cycles with reproducible switching of the ionic response are shown in Figure 7c, highlighting the robustness and reversibility of the pH-controlled assembly [43].

#### 2.2.2. Light

**Figure 8:**
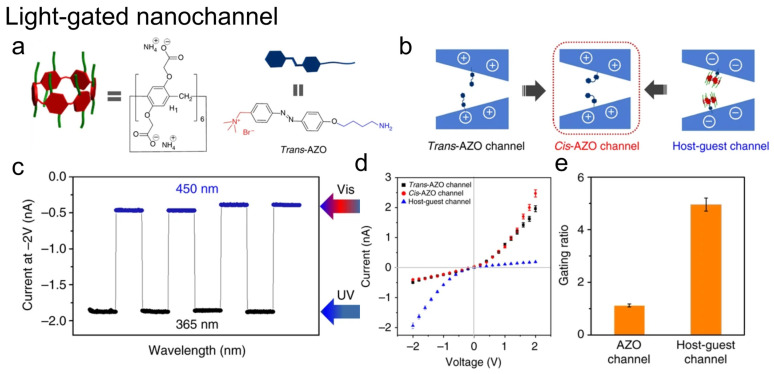
Light-gated nanochannel based on azobenzene host–guest interactions. (**a**) Chemical structures of pillar[6]arene (P6A) and trans-AZO. (**b**) Schematic of the trans-AZO channel, cis-AZO channel, and the assembled host–guest channel onto trans-AZO configuration. (**c**) Reversible current switching at −2 V under alternating UV (365 nm) and visible (450 nm) irradiation. (**d**) I–V curves of the trans-AZO, cis-AZO, and host–guest channels. (**e**) Gating ratio comparison for the AZO channel and the host–guest channel. Adapted from Ref. [44].

Light-responsive host–guest platforms have been developed using biological channel rhodopsins as inspiration. Sun et al. engineered nanochannels incorporating negatively charged pillar[6]arene (P6A) and positively charged azobenzene (AZO) [44]. UV/visible light-induced trans-cis isomerization drives reversible assembly/disassembly, switching between cation-selective and anion-selective states. Nanochannels were fabricated by grafting AZO groups onto etched PET membranes followed by P6A self-assembly. The modified channels showed a gating ratio of ~5, markedly higher than AZO-only channels (Figure 8) [44].

Following a similar strategy, Quan et al. constructed a visible-light-regulated chloride-transport membrane channel [45]. Instead of azobenzene, a natural retinal chromophore was covalently grafted into the conical PET nanochannels and used as the photochromic guest, while an ethyl-urea-functionalized pillar[6]arene (Urea-P6) acted as a Cl^−^-binding host that assembles onto the retinal-modified surface. Visible-light-induced trans-cis isomerization of retinal disrupts this host–guest pair, releasing Urea-P6 and decreasing the local Cl^−^-binding sites, which switches the channel between high- and low-conductance states for chloride in a reversible fashion [45].

Similarly, Liu et al. employed reversible interactions between β-cyclodextrin (β-CD) and azobenzene to enable bidirectional ion rectification in PI nanochannels [46]. Asymmetric functionalization of opposite channel surfaces with Azo-NH_2_ and Azo-COOH, combined with pH and light regulation, enabled dynamic switching between forward and backward rectification. Backward rectification occurred at pH < 4, while forward rectification dominated at pH > 7 [46].

#### 2.2.3. Temperature

**Figure 9:**
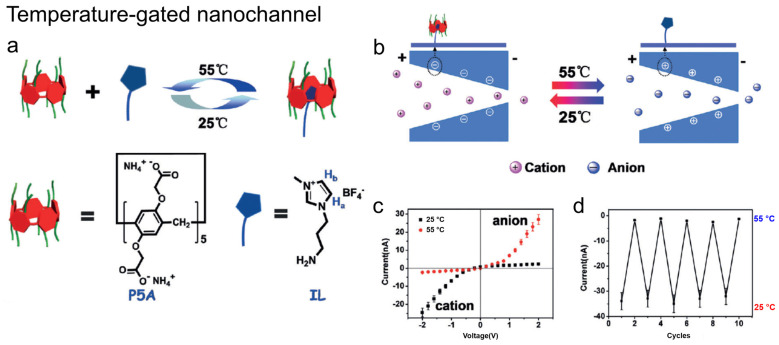
(**a**) Temperature-controlled host–guest binding between pillar[5]arene (P5A) and IL. (**b**) Schematic of temperature-dependent ion transport in the modified nanochannel. (**c**) I–V curves at 25 °C and 55 °C. (**d**) Reversible current switching upon temperature cycling (25–55 °C). Adapted from Ref. [47].

Temperature has also been investigated as a stimulus. Li and colleagues integrated pillar[5]arene (P5A) with ionic liquid (IL) guest molecules to yield thermoresponsive nanochannels in which P5A-IL complexation reversibly assembles/disassembles with temperature changes. At 25 °C, cation-selective transport is observed, whereas at 55 °C, anion selectivity emerges. These transitions are reversible, highlighting the potential for biomimetic thermal regulation in nanofluidics (Figure 9) [47].

Temperature can also be used to actuate host–guest gating for controlled molecular release. For instance, Qu et al. reported dynamically regulated biomimetic nanochannels inspired by temperature-sensitive proteins (CorA), where a temperature-responsive host–guest supramolecular assembly confined within the channels provides multiple tunable gating states and good cycling stability, enabling temperature-programmed ‘intelligent’ release across different pesticides [48].

#### 2.2.4. Medium Redox State

**Figure 10:**
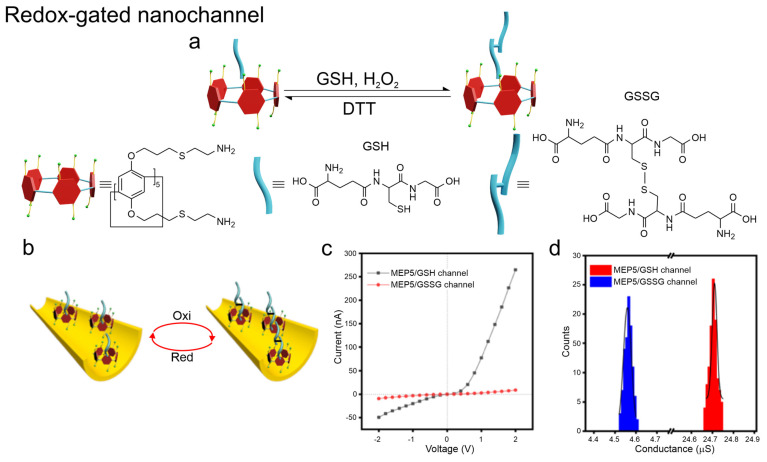
Redox-gated nanochannel based on reversible MEP5–GSH/GSSG host–guest interactions. (**a**) Redox switching between GSH and GSSG controlled by H_2_O_2_ and DTT and corresponding host–guest states. (**b**) Schematic of oxidation/reduction-driven transport modulation in the nanochannel. (**c**) I–V curves for the MEP5/GSH and MEP5/GSSG channels. (**d**) Conductance distribution (counts) for both states. Adapted from Ref. [49].

Analogously to biological ion channels, host–guest architectures can also be designed so that their conductance states are regulated by the redox state of the surrounding medium. In cellular environments, a widely used indicator of the redox state is glutathione (GSH), in particular the ratio between its reduced (GSH) and oxidized (GSSG) forms. As a representative example, PET nanochannels functionalized with mercaptoethylamine pillar[5]arene (MEP5) switch between conductive and nonconductive states depending on the redox state of GSH. Binding of reduced GSH increases positive charge density (“on” state), while oxidation to GSSG disrupts binding (“off” state) (Figure 10). The process is reversible upon reduction by dithiothreitol (DTT). Strong selectivity was confirmed by calculated binding energies of −161.18 kJ·mol^−1^ (GSH) vs. −92.06 kJ·mol^−1^ (GSSG). Excellent recyclability was demonstrated over multiple redox cycles [49].

## 3. Future Perspectives

A clearer trajectory is emerging for chemically functionalized nano/microchannel membranes: progressing from compelling proof-of-concept demonstrations toward modular, scalable platforms in which molecular recognition is coupled to an iontronic readout in a repeatable, calibratable, and robust manner. From this perspective, future opportunities naturally cluster into three directions: chemical and biosensing, where host–guest binding or catalytic events are encoded into distinctive iontronic signatures; selective transport, separations, and energy conversion, where rectification and ion-selective pathways can be leveraged under realistic gradients and mixed electrolytes; and iontronic device concepts, in which switchable transport states are engineered into transistor-like, memory, or logic-like behaviors [6].

Regarding ion sensing, a realistic near-term trajectory is a shift from discrete, end-point concentration readouts toward continuous, real-time monitoring, leveraging ionic current rectification and gating as intrinsic amplification steps that convert molecular recognition into robust iontronic signatures. The next leap is not just lower limit of detection (LoD), but deployability: integrating these sensors into lab-on-a-chip microfluidics (fixed electrodes, tiny sample volumes, controlled flow) and scaling to multichannel/parallel formats to enable multiplexed ion panels and faster, more robust readouts [50].

In the case of selective ion sieving, a near-term direction is to tune ionic composition, which has strong translational relevance in the treatment of, for example, brackish waters and brines. One practical advantage is the selective suppression of Mg^+2^/Ca^+2^ to mitigate mineral scaling and improve the performance of downstream separation processes [51]. In this context, crown-ether confinement within UiO-66 illustrates how coupling rigid nanoconfinement with specific coordination can yield pronounced mono/divalent discrimination (e.g., K^+^/Mg^+2^), providing mechanistic design principles that are transferable to other selective-membrane architectures [52].

Moreover, host–guest chemistry provides a natural entry point to iontronic logic and information processing because molecular recognition can be mapped onto discrete conductance/rectification states that function as inputs, thresholds, and readouts in the ionic domain. Recent host–guest nanochannel demonstrations of multilevel encryption underscore this broader potential: beyond sensing, nanofluidic devices can be designed to encode, route, and transform chemical inputs into rule-based ionic outputs, enabling logic-like functionality directly in wet, ion-rich environments where lab-on-a-chip platforms naturally operate [41].

Across all three approaches, the emphasis is shifting from merely demonstrating proof-of-concept responses toward achieving well-defined and reproducible performance across relevant operating windows, supported by comparable figures of merit, long-term stability, and device-to-device reproducibility. To date, these aspects have been only superficially addressed in single track-etched nanochannel platforms. Reported results indicate that the complexity of realistic electrolytic media, together with variability arising from nanochannel fabrication and supramolecular functionalization, requires further optimization before integration into practically applicable devices. In addition, storage stability and operational durability remain critical challenges, as evidence suggests that many systems still exhibit limited lifetimes under continuous operation or even under storage conditions. In this scenario, several improvement strategies could be envisioned to address these limitations. For instance, the incorporation of additional protective layers directly onto the nanochannel surface may enhance the stability of the supramolecular assembly under prolonged operation. Alternatively, upstream pretreatment strategies such as the integration of filtration membranes or absorbent materials within the fluidic pathway could serve as effective sample preconditioning steps, minimizing fouling, degradation, or blockage of the nanochannels and preserving the integrity of their supramolecular functionalization.

## 4. Conclusions

This review presented studies on the selective sensing and transport of ions and chiral molecules using macrocyclic host–guest surface-modified single track-etched nanochannels, in which guest recognition events act as chemical gating elements. The systems are reviewed according to host structure and externally controlled functions. In these architectures, guest recognition depends on the cavity size and functional groups of the macrocyclic host. Crown ethers primarily act as hosts for alkali metal ions, whereas calixarenes and pillararenes extend selectivity toward group IIA/IIB metal cations and group VIIA halide anions. In parallel, macrocyclic hosts based on chiral chemistry exhibit enantioselective recognition toward a wide range of chiral guests.

The reviewed examples demonstrate that incorporating host molecules into nanofluidic architectures, in combination with iontronic readout strategies, provides a robust pathway toward the development of ion-selective and stimulus-responsive platforms with biologically comparable selectivity and nanomolar detection limits. This capability is particularly significant in sensing applications, where many of the ionic analytes discussed lack intrinsic redox activity or measurable UV–Vis absorption, rendering conventional transduction approaches inherently challenging. In this context, iontronics becomes indispensable, as it enables the conversion of subtle physicochemical events associated with host–guest complexation into quantifiable electrical signals.

Beyond sensing applications, host–guest interactions within nanofluidic environments have recently been exploited to engineer membranes with finely tuned ion-sieving properties [52,53,54,55,56]. In such systems, nanochannel-containing membranes are functionalized with host molecules such as crown ethers, thereby enabling the selective and rapid transport of target ions. Taken together, these studies position the convergence of supramolecular host–guest chemistry, nanofluidic architectures, and iontronic transduction as a compelling paradigm for applications in sensing and ion sieving, as well as a complementary strategy for ion removal and desalination processes.

## Data Availability

No new data were created or analyzed in this study. Data sharing is not applicable to this article.

## Abbreviations

The following abbreviations are used in this manuscript:

FET	field-effect transistor
ISFET	ion-sensitive field-effect transistor
CHEMFET	chemically sensitive field-effect transistor
DO3A	1,4,7,10-tetraazacyclododecane-1,4,7-triacetic acid
PI	polyimide
4-AB15C5	4′-aminobenzo-15-crown-5
4-AB18C6	4′-aminobenzo-18-crown-6
NMR	nuclear magnetic resonance
PNP	Poisson–Nernst–Planck
MOF	Metal–Organic Framework
PET	polyethylene terephthalate
MOFSNC	MOF subnanochannel
C4CE	dipropargylaza-p-tert-butyl calix[4]crown ether
XPS	X-ray photoelectron spectroscopy
LSCM	laser scanning confocal microscopy
t-BuC[4]C	p-tert-butylcalix[4]arene-crown
MAP5	mercaptoacetic acid pillar[5]arene
HDA	1,6-hexanediamine
NP5	NH2-pillar[5]arene
AZO	azobenzene
P6A	pillar[6]arene
Urea-P6	ethyl-urea-functionalized pillar[6]arene
β-CD	β-cyclodextrin
Azo-NH2	amino-functionalized azobenzene
Azo-COOH	carboxylic-functionalized azobenzene
P5A	pillar[5]arene
IL	ionic liquid
P5A-IL	pillar[5]arene–ionic liquid hybrid
DTPA	diethylenetriaminepentaacetic acid
GSH	reduced glutathione
GSSG	oxidized glutathione
MEP5	mercaptoethylamine pillar[5]arene
DTT	dithiothreitol
LoD	limit of detection
γ-CD	γ-cyclodextrin
NEDA	N-(1-naphthyl)ethylenediamine
